# PhytoPipe: a phytosanitary pipeline for plant pathogen detection and diagnosis using RNA-seq data

**DOI:** 10.1186/s12859-023-05589-2

**Published:** 2023-12-13

**Authors:** Xiaojun Hu, Oscar P. Hurtado-Gonzales, Bishwo N. Adhikari, Ronald D. French-Monar, Martha Malapi, Joseph A. Foster, Clint D. McFarland

**Affiliations:** 1 United States Department of Agriculture (USDA), Animal and Plant Health Inspection Service (APHIS), Plant Protection and Quarantine (PPQ), Plant Germplasm Quarantine Program (PGQP), Beltsville, MD USA; 2 Present Address: American Seed Trade Association (ASTA), Alexandria, VA USA; 3 USDA-APHIS-PPQ, Field Operations, Raleigh, NC USA

**Keywords:** Phytopathogen, Pipeline, Phytosanitary, Quarantine, Pathogen detection, Bioinformatics, HTS, RNA-seq

## Abstract

**Background:**

Detection of exotic plant pathogens and preventing their entry and establishment are critical for the protection of agricultural systems while securing the global trading of agricultural commodities. High-throughput sequencing (HTS) has been applied successfully for plant pathogen discovery, leading to its current application in routine pathogen detection. However, the analysis of massive amounts of HTS data has become one of the major challenges for the use of HTS more broadly as a rapid diagnostics tool. Several bioinformatics pipelines have been developed to handle HTS data with a focus on plant virus and viroid detection. However, there is a need for an integrative tool that can simultaneously detect a wider range of other plant pathogens in HTS data, such as bacteria (including phytoplasmas), fungi, and oomycetes, and this tool should also be capable of generating a comprehensive report on the phytosanitary status of the diagnosed specimen.

**Results:**

We have developed an open-source bioinformatics pipeline called PhytoPipe (Phytosanitary Pipeline) to provide the plant pathology diagnostician community with a user-friendly tool that integrates analysis and visualization of HTS RNA-seq data. PhytoPipe includes quality control of reads, read classification, assembly-based annotation, and reference-based mapping. The final product of the analysis is a comprehensive report for easy interpretation of not only viruses and viroids but also bacteria (including phytoplasma), fungi, and oomycetes. PhytoPipe is implemented in Snakemake workflow with Python 3 and bash scripts in a Linux environment. The source code for PhytoPipe is freely available and distributed under a BSD-3 license.

**Conclusions:**

PhytoPipe provides an integrative bioinformatics pipeline that can be used for the analysis of HTS RNA-seq data. PhytoPipe is easily installed on a Linux or Mac system and can be conveniently used with a Docker image, which includes all dependent packages and software related to analyses. It is publicly available on GitHub at https://github.com/healthyPlant/PhytoPipe and on Docker Hub at https://hub.docker.com/r/healthyplant/phytopipe.

**Supplementary Information:**

The online version contains supplementary material available at 10.1186/s12859-023-05589-2.

## Background

International trade and consumer demand have increased the worldwide movement of plants and plant parts. At the same time, the global distribution and exchange of plant germplasm that support the improvement and expansion of agricultural and horticultural industries have also grown dramatically in recent years [1–4]. Imported plant germplasm must be thoroughly tested, and proper phytosanitary measures should be followed to minimize the risk of introduction of new pests and pathogens of quarantine relevance. Therefore, the development of comprehensive diagnostic methods to identify both known and unknown plant pathogens, as well as novel variants, is an important goal for testing plant material distributed at the global and national levels.

Quarantine centers, certification programs, and plant diagnostic clinics have been using traditional virus diagnostic techniques to conduct virus detection, which includes biological indexing (mechanical transmission using herbaceous indicator plants such as *Chenopodium quinoa* and *Nicotiana tabacum*, etc.), enzyme-linked immunosorbent assay (ELISA), polymerase chain reaction (PCR), and loop-mediated isothermal amplification (LAMP) [5, 6]. More recently, high-throughput sequencing (HTS), also known as next-generation sequencing (NGS) or deep sequencing has been used by diagnosticians and researchers for detecting and identifying plant pathogens, which has resulted in a steady increase in the identification of plant pathogens affecting various crops [2, 7–13]. Since it does not require a priori phytosanitary status knowledge of the sample, HTS offers certain advantages when compared to targeted-diagnostic techniques such as ELISA or PCR [10, 11, 14–18]. The use of HTS is now becoming a gold standard across continents after the International Plant Protection Convention (IPPC) recommended it as a diagnostic tool for phytosanitary purposes in 2019 [19]. More recently, the European and Mediterranean Plant Protection Organization (EPPO) released a standard for plant health diagnostics using HTS in 2022 [20].

HTS-based plant pathogen detection involves two major strategies: amplicon sequencing, which uses the power of PCR to amplify specific standardized genetic marker(s), such as 16S rRNA gene for bacteria [21] or unique genomic regions of virus and viroid genomes [22]; or shotgun sequencing, which captures the complete nucleic acids present in a sample [20, 23]. Amplicon sequencing is popularly used for identification and comparison of entire microbial communities while shotgun sequencing has wider applications for uncovering novel and emerging pathogens [23]. Currently, total RNA sequencing (RNA-seq) and small RNA sequencing (sRNA-seq) are the two most widely used HTS shotgun approaches for the detection of plant viruses and viroids [27, 28]. sRNA-seq is designed for virus detection based on the plant viral response mechanism [29] while total RNA-seq, which had traditionally been used for the analysis of the transcriptomic landscape of the host, is now also used for the detection of plant pathogens such as bacteria (including phytoplasma), fungi, viruses, and viroids [8, 30–32]. Although RNA-seq is commonly used for virus and viroid detection by plant virologists and in routine diagnostic applications such as post-entry quarantine or certification [11, 33], non-viral pathogens or pests can also be detected from the same RNA-seq dataset used for virus detection [15]. Because of its broad detection spectrum and novel pathogen detection ability, it has recently become an increasingly popular tool for pathogen detection. It has been used in many crops, such as wheat [34], grapevine [35], citrus [36], fruit trees [37], cucurbit [38], sugarcane [10], grasses [7, 38] etc. for viral pathogen detection.

One of the drawbacks of the RNA-seq approach is that it requires higher titer levels of the pathogen expressed in the hosts, which corresponds to a high number of pathogen-derived reads in the data [15]. The RNA-seq approach could miss pathogens with low titer. Another obstacle for using RNA-seq to detect pathogens is its demanding bioinformatic requirements for microbiome analysis. To our knowledge, there are few broadly accepted standard methods for RNA-seq data generation, processing, and analysis [25, 39, 40]. The most used analysis methods or pipelines can fall into three main categories: (i) mapping sequence reads directly to reference genomes from known pathogens such as Pathoscope [41] and CAMAMED [42]; (ii) assembling sequence reads and annotating contigs such as VirFind [43], VSD toolkit [44], VirusDetect [45], and Virtool [37]; and (iii) read-based taxonomic assignments such as Kaiju [46], Kraken2 [47], and Kodoja [48]. However, these methods or pipelines do not offer an integrated sequence read quality control, read assembly, pathogen reference mapping, and read classification to identify known pathogens and discover novel species, which is a common occurrence during plant virus detection. Therefore, an integrative and comprehensive pipeline is needed to detect the presence of a wide range of potential plant pathogens in a specimen.

Here we present Phytosanitary Pipeline (PhytoPipe), an integrative pipeline for plant pathogen identification using RNA-seq data. The pipeline combines current tools for HTS read quality control, the host read filtering, read assembly, contig annotation, reference mapping, and taxonomic classification. PhytoPipe is equally capable of identifying known bacteria (including phytoplasma), fungi, oomycetes, viruses, viroids, and possible novel viruses. Furthermore, the use of the Snakemake workflow management system [49] allows for an efficient and automated deployment on a local multicore computer, computing cluster, or a cloud environment.

## Implementation

**Fig. 1:**
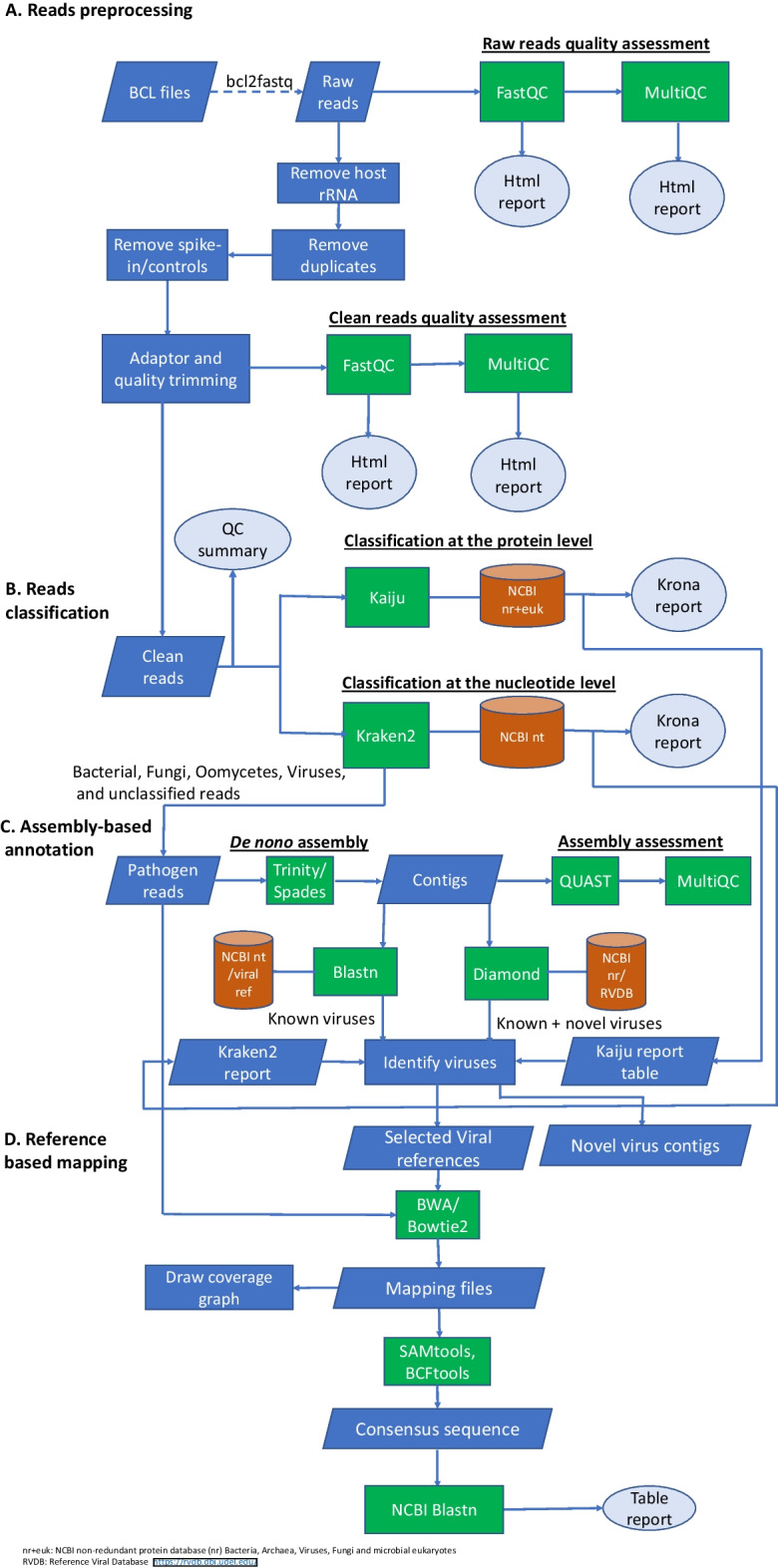
Flowchart describing processes and steps performed by the PhytoPipe workflow. The pipeline integrates reads preprocessing (**A**), reads classification (**B**), assembly-based annotation (**C**), and reference-based mapping (**D**) into a single workflow. The entire protocol can be run starting from raw sequence data in the form of single- or paired-end FASTQ files. The results are summarized in an HTML report file, tables, and Krona plots for an expert interpretation

**Fig. 2:**
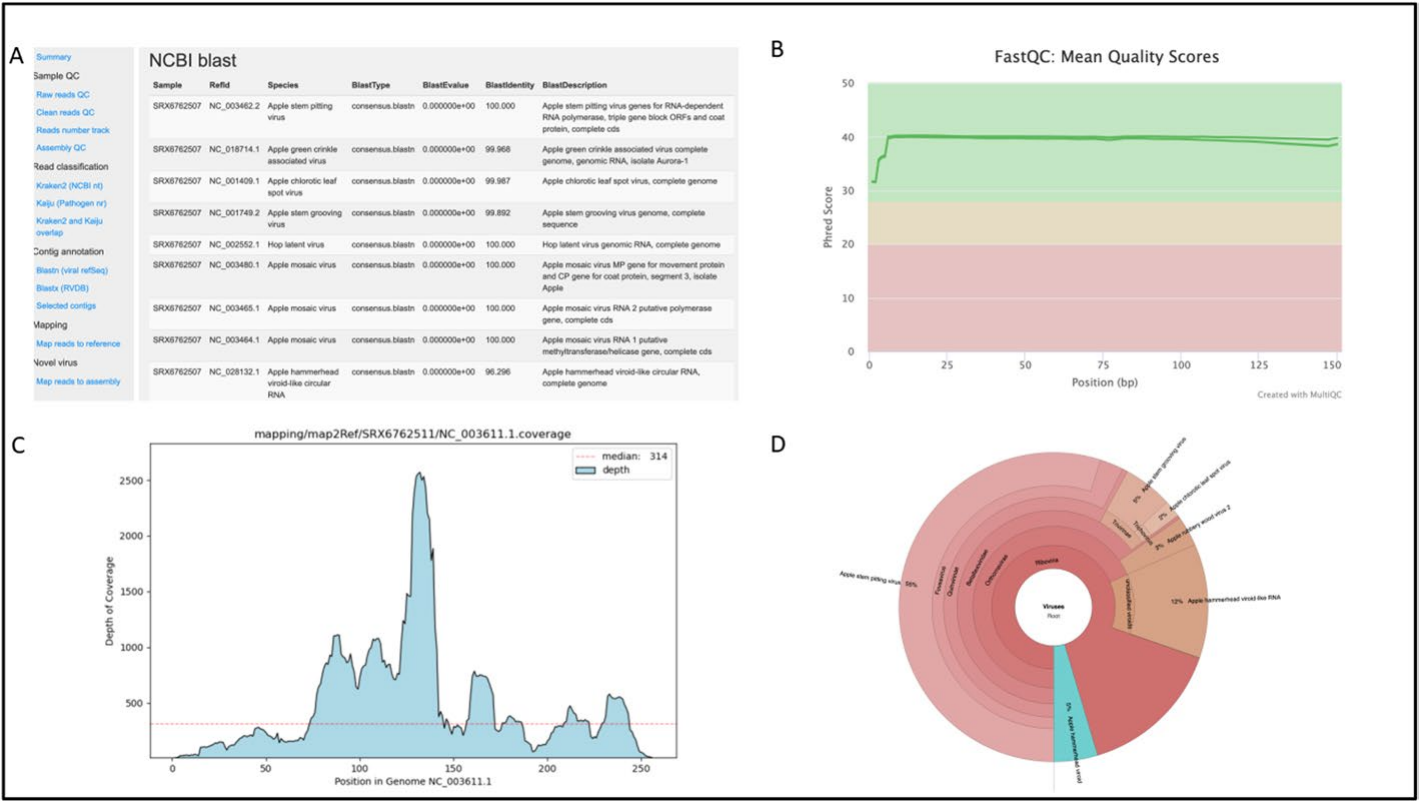
Example output from the PhytoPipe workflow. RNA-seq data from two apple samples (NCBI BioProject accession: PRJNA562540 [72]) processed by PhytoPipe show: **A** an HTML report showing results from different methods; **B** a MultiQC report for samples read quality; **C** a read mapping graph for a virus; **D** a Krona pie graph showing the taxonomic composition

The PhytoPipe framework uses the Snakemake workflow management system [49] to organize sequence data processing tools. These tools have been organized into four distinct modules: reads preprocessing (Fig. 1A), reads classification (Fig. 1B), assembly-based annotation (Fig. 1C), and reference-based mapping (Fig. 1D). Each module summarizes the results into HTML or Krona reports and tables (Fig. 2). PhytoPipe can be easily set up in a Linux or Mac environment or run using the PhytoPipe docker image [50] (https://hub.docker.com/r/healthyplant/phytopipe) on a Linux, Mac, or Windows system. PhytoPipe requires at least 300 GB of RAM, 1 TB of fast-speed storage and multi-cores (> 32) parallel computing environment. The complete usage and user options are outlined on the GitHub wiki page https://github.com/healthyPlant/PhytoPipe/wiki.

### Quality control

PhytoPipe can process sequence reads in the form of single- or paired-end FASTQ files and performs raw sequence reads cleaning in four steps: (1) removing host ribosomal RNAs (rRNA) with *bbduk* against the SILVA eukaryote ribosomal 18S and 28S RNA database [51], which is summarized by SortMeRNA [52]; (2) removing PCR duplicates with *clumpify*; (3) removing spike-in or positive controls (the default is pre-determined as PhiX) with *BBSplit*; and (4) removing and trimming low-quality reads, bases, and adapter sequences with Trimmomatic [53] (Fig. 1A). The tools used in steps 1, 2, and 3 are implemented in the BBTools suite [54]. The raw and clean-read qualities for a single sample are visualized by FastQC [55] and for batch samples by MultiQC [56]. PhytoPipe reports read numbers at each cleaning step, so the user can choose them to evaluate the wet lab work, such as rRNA depletion efficiency.

### Read classification

PhytoPipe uses Kraken2 [47] to query reads against the NCBI nt database for the nucleotide-level classification. PhytoPipe also relies on Kaiju [46] to assign reads to taxa using the NCBI taxonomy and a microbial non-redundant database (nr + euk) of bacterial, viral, fungal, and other microbial eukaryotic proteins (Fig. 1B). These *k*-mer-based approaches classify sequences based on the presence and frequency of specific *k*-mers in the database [46, 57, 58]. They can discover low titer viruses or phytoplasma, which could be missed by assembly-based methods [29]. Besides the text report generated by the tools, the sequence profile and the metagenomic classification are also interactively visualized by multi-layered Krona pie charts for a given sample [59].

### Assembly-based annotation

Prior to read assembly, host reads are usually subtracted for pathogen detection. Instead of mapping reads to the genome to remove host reads, PhytoPipe extracts possible pathogen-derived reads, including classified pathogen-derived (bacteria, fungi, oomycetes, viruses, and viroids) and unclassified reads from the Kraken2 classification using the modified script “extract_kraken_reads.py” in KrakenTools [60] (Fig. 1C). Then these reads are assembled with either SPAdes [61] or Trinity [62] de novo assembler. Trinity is used as default due to its robustness and its ability to better perform when dealing with low titer viruses. Assemblies are then evaluated with QUAST [63] followed by contig (length ≥ 200 nucleotides) annotation at the nucleotide level using blastn [64] against NCBI nt database and at the protein level using Diamond blastx [65] against NCBI nr database. PhytoPipe allows users to obtain the pathogen information in the blast results that are combined with pathogen taxonomy along with HTS read count assigned by Kraken2 and Kaiju. Blast searches against NCBI databases can be time-consuming (several days) depending on the user’s computing environment and the volume of the HTS data. Hence, the user who is just interested in the virus discovery can either use their own database or other alternative virus databases such as NCBI viral reference genomes and Reference Viral Databases (RVDB) protein version [66]. The user’s databases and the analysis parameters can be easily set up in the config file. Finally, the users could identify possible novel viruses based on Diamond blastx results and the ICTV criteria field [67].

### Reference-based mapping

To further confirm virus discovery derived from HTS read classification and assembly-based annotation, viral reference genomes are collated before reference-based mapping by PhytoPipe (Fig. 1D). The clean reads are mapped to reference genomes by BWA-MEM [68]. The mapped read number and coverage are calculated by SAMtools [69] and a coverage graph is drawn using matplotlib [70] in Python. A consensus sequence is generated with BCFtools (including mpileup and consensus commands) [71] and is additionally annotated using blastn against the local NCBI nt database to filter non-pathogen sequences.

## Results

The PhytoPipe output for each sample includes FastQC/MultiQC reports for HTS read quality assessment, Krona taxonomy pie charts for both Kraken2/Kaiju read classification and blastn/blastx results for contigs, and QUAST report for assembly evaluation. In addition, output also includes blastn/Diamond blastx search result tables, mapping statistics and coverage graphs for viruses and viroids, together with a summary report in HTML format (report.html). The final text report (report.txt) for viruses and viroids includes results from all samples, including the raw/clean-read length and count, read mapping information (reference names and related taxonomy, mapped read count, normalized read count (reads per kilobase of transcript per million mapped reads (RPKM)), percentage of mapped reads, percentage of viral genome covered, and mean coverage), NCBI blast results (blast E-value, blast identity, blast description), and the nucleotide sequence (contig or consensus sequence). A comprehensive sequence quality report includes a read quality table of raw read count, raw bases (Mbases) count, percentage of bases >  = Q30, mean of raw read quality score, percentage of rRNA, read count after removing duplicates, spike-in/control read count, read count after trimming, and the number of possible pathogen-derived reads used for assembly.

To show the PhytoPipe detection of microbes in the real plant RNA-seq data, two apple sample datasets from the study by Wright et al. [72] (host: *Malus domestica*, NCBI BioProject accession: PRJNA562540) were analyzed. Table 1 shows the microbe detection results. One fungus (*Aureobasidium pullulans* EXF-150) was found in SRX6762507, which could have been derived from the environment (e.g., water or soil). Fungal viruses (also known as mycoviruses) were also found in this sample (not listed). Three species of bacteria (*Actinoplanes friuliensis* DSM 7358, *Bradyrhizobium sp.* 170, and *Steroidobacter denitrificans*) were found in SRX6762511, which could be soilborne. The validated multiple apple viruses and viroids were also found by PhytoPipe in both samples: apple chlorotic leaf spot virus (ACLSV), apple hammerhead viroid (AHVd), apple mosaic virus (ApMV), apple rubbery wood virus 2 (ARWV2), apple stem grooving virus (ASGV), apple stem pitting virus (ASPV). PhytoPipe also found additional three ones: hop latent virus, hop latent viroid, and hop stunt viroid. Besides this output for all microbes, PhytoPipe has a specific report for viruses and viroids (report.txt), the Table 1 missed validated virus, apple green crinkle associated virus (AGCaV), is in the viral report. Figure 2 shows examples of the PhytoPipe output from this analysis. The HTML report contains summary results from different tools (Fig. 2A), the MultiQC report shows read quality (Fig. 2B), the read mapping graph shows genome coverage for virus and viroid genomes (in this case hop latent viroid: NC_003611) (Fig. 2C), and a Krona pie chart shows the taxonomic composition of the sample (Fig. 2D). The Krona pie chart also offers an interactive view of different pathogens present in the sample. Details of these result files are provided on GitHub (https://github.com/healthyPlant/PhytoPipe/tree/main/doc/test_report.zip).

**Table 1:** Microbes and pathogens detected by PhytoPipe in samples SRX6762507 and SRX6762511

Sample	Types of microbes	Microbe name	Read number by classification	Contig number	Longest Contig size
SRX6762507 (lL)^b^	Fungi	*Aureobasidium pullulans* EXF-150	87747	6149	6444
	Viruses	apple stem pitting virus	8552	135	9192
	Viruses	apple chlorotic leaf spot virus	7315	10	7555
	Viruses	apple stem grooving virus	4226	1	6488
	Viruses	hop latent virus	278	3	4391
	Viruses	apple mosaic virus	233	8	1992
	Viruses	apple rubbery wood virus 2	104	7	434
	Viroids	hop stunt viroid	47	1	390
	Viroids	hop latent viroid	18	1	347
	Viroids	apple hammerhead viroid-like RNA	12	1	452
SRX6762511 (1R)^b^	Bacteria	*Actinoplanes friuliensis DSM 7358*	16231	1732	6952
	Bacteria	*Bradyrhizobium sp.* 170	13597	3016	13027
	Bacteria	*Steroidobacter denitrificans*	12222	934	9892
	Viruses	apple stem pitting virus	80565	58	9356
	Viruses	apple hammerhead viroid	10059	2	1207^a^
	Viruses	apple stem grooving virus	4570	1	6482
	Viruses	apple rubbery wood virus 2	2694	9	1626
	Viruses	apple chlorotic leaf spot virus	1670	21	7565
	Viruses	hop latent virus	357	3	5364
	Viruses	apple mosaic virus	297	9	1996
	Viroids	hop stunt viroid	44	1	390
	Viroids	hop latent viroid	8	1	342

^a^1207 nt is not an expected viroid size. It’s an assembly error

^b^Sample name in the study

To compare PhytoPipe with other plant virus detection pipelines, nine datasets corresponding to nine virus detection challenges from the Plant Health Bioinformatics Network (PHBN) VIROMOCK (https://gitlab.com/ilvo/VIROMOCK-challenge) [73] were analyzed. Dataset_2 was excluded since it was designed for mutation detection. Table 2 shows the results of the virus and viroid detection using four different pipelines. PhytoPipe could detect all expected viruses and viroids in the datasets and solved all the pre-determined challenges listed for these datasets. Pipelines Kodoja [29], Pathoscope [41], and Virtool [37] detected most of the known viruses at the species level but missed one to five viruses. Both Kodaja and Pathoscope failed to detect novel viruses. Virtool, on the other hand, could detect novel viruses and solve several challenges. True positive rate (TPR), false negative rate (FNR), and false discovery rate (FDR) are calculated by true positives (TP) (detected expected viruses), false positives (FP) (detected unexpected viruses), and false negatives (FN) (missed expected viruses). TPR = TP/(TP + FN), FNR = FN/(TP + FN) and FDR = FP/(FP + TP). The TPRs for the four pipelines are 100% (PhytoPipe) > 91% (Pathoscope) > 74% (Virtool) > 52% (Kodoja); The FNRs are 0% (PhytoPipe) < 9% (Pathoscope) < 26% (Virtool) < 48% (Kodoja); The FDRs are 39% (PhytoPipe) > 25% (Kodoja) > 22% (Pathoscope) > 19% (Virtool). PhytoPipe has the highest TPR and lowest FNR. It has the highest FDR since some of the identified viruses are not in the expected virus list. For example, citrus blight-associated pararetrovirus, citrus endogenous pararetrovirus, and cherry virus A in the dataset_1 (citrus sample); grapevine fleck virus, grapevine leafroll-associated virus 3, grapevine Kizil Sapak virus, and grapevine leafroll-associated virus 7 in the dataset_3 (grapevine sample); pistacia cryptic virus in the dataset_9 (pistachio sample). To determine whether they are true or false positives, confirmatory experiments such as the use of a PCR-based detection method might be necessary.

**Table 2:** Comparison of virus and viroid detection with different pipelines

	Expected viruses and viroids	Challenge	PhytoPipe		Kodoja^a^		Pathoscope^b^		Virtool	
			Detection (detected/all)	Challenge finish status	Detection (detected/all)	Challenge finish status	Detection (detected/all)	Challenge finish status	Detection (detected/all)	Challenge finish status
Dataset_1	CTV, CVEV, CEVd, CVd-III, HSVd	Different viral concentration (CTV)	5/5	Found different CTV strains	2/5 (CEVd, CVd-III, HSVd missed)	Not applicable	5/5	Not applicable	5/5	Not applicable
Dataset_3	GRSPaV, GLRaV2, GRVFV, HSVd, GYSVd-1	Different viral concentration (at the species level) + Non complete virus genome coverage (GRSPaV, GLRaV2 and GRVFV)	5/5	Viruses at the species level were found	3/5 (HSVd, GYSVd-1 missed)	Not applicable	4/5 (GRVFV missed)	Not applicable	2/5 (GRSPaV, GRVFV, GYSVd-1 missed)	Viruses with non-complete genome could be missed
Dataset_4	GRBV, GRSPaV, HSVd, GYSVd-1, GYSVd-2	Very similar viroids sequence (GYSVd-1 and GYSVd-2)	5/5	Two similar viroids were found	2/5 (HSVd, GYSVd-1, GYSVd-2 missed)	Not applicable	5/5	Not applicable	4/5	Can’t separate GYSVd-1 and -2
Dataset_5	PVY NTN and N strains	Mix of recombinant and parental PVY strains	2/2	Different PVY strains and recombinants were detected	1/1	Not applicable	1/1	Not applicable	1/1	Recombinant NTN detected, not parental strains
Dataset_6	PVY	New PVY strain	2/2	New PVY strain detected	1/1	Not applicable	1/1	Not applicable	2/2	New PVY strain detected
Dataset_7	TSWV	Complete genome + defective form of TSWV	2/2	A defective L segment of TSWV was detected	1/1	Not applicable	1/1	Not applicable	2/2	A defective L segment of TSWV was detected
Dataset_8	PFBV, CqMV1	Cryptic mitovirus virus + low mitovirus concentration	2/2	A low concentration CqMV1 was detected	1/2 (CqMV1 missed)	Not applicable	2/2	Not applicable	1/2 (CqMV1 missed)	Mitovirus missed
Dataset_9	PiVB	Concentration of different PiVB genomic segments	1/1	All eight PiVB segments were detected	0/1 (PiVB missed)	Not applicable	1/1	Not applicable	0/1 (PiVB missed)	Not applicable
Dataset_10	PBNSPaV, PPV	New PPV strain	2/2	New PPV strain was detected	1/2 (PPV missed)	Not applicable	1/2 (PPV missed)	Not applicable	2/2	New PPV strain was detected

Virus and viroid abbreviations: citrus dwarfing viroid (CDVd) (former name citrus viroid III (CVd-III)), citrus vein enation virus (CVEV); citrus exocortis viroid (CEVd), hop stunt viroid (HSVd), citrus tristeza virus (CTV), grapevine rupestris vein feathering virus (GRVFV), grapevine rupestris stem pitting-associated virus (GRSPaV), grapevine leafroll-associated virus 2 (GLRaV2), grapevine yellow speckle viroid 1 (GYSVd1), grapevine red blotch virus (GRBV),, grapevine yellow speckle viroid 2 (GYSVd-2), potato virus Y (PVY), tomato spotted wilt virus (TSWV), pelargonium flower break virus (PFBV), chenopodium quinoa mitovirus 1 (CqMV1), pistacia emaravirus B (PiVB), plum bark necrosis stem pitting-associated virus (PBNSPaV), plum pox virus (PPV)

^a^Used “Species sequences (stringent)” result with read number > 10 as a threshold. Viroids are filtered in the stringent result

^b^Used “GenomeCoverageMax” > 25% as a threshold

To demonstrate the capabilities of PhytoPipe to detect bacteria, fungi, and oomycetes, twenty-two public RNA-seq sequence datasets (twelve bacteria, eight fungi, and two oomycetes) from the pathogen infected plants in the study by Haegeman et al. in 2023 [15] were analyzed using PhytoPipe. The results in Table 3 show that PhytoPipe detected confirmed pathogens in 18 out of 22 (81.8%) samples. PhytoPipe was not able to detect bacteria from four samples due to the low abundance of the pathogen-derived reads (reads per million reads(rpm) < 10 as reported by Haegeman et al.).

**Table 3:** PhytoPipe analyses results for RNA-seq samples with confirmed pathogen infection

Sample ID/accession	Host plant	Confirmed pathogen infection	PhytoPipe detection	Taxon Observed in PhytoPipe	Contig numbers	Longest contig size	Kraken2 classified read number
SRR5100668	*Solanum tuberosum* (potato)	*Ca*. Liberibacter solanacearum (bacterium)	No	NA	NA	NA	NA
SRR10148792	*Citrus sinensis* (orange)	*Ca*. Liberibacter asiaticus (bacterium)	Yes	*Candidatus* Liberibacter asiaticus	6	1799	235
SRR8295844	*Citrus sinensis* (orange)	*Ca*. Liberibacter asiaticus (bacterium)	Yes	*Candidatus* Liberibacter asiaticus	4	743	1289
SRR9225242	*Solanum lycopersicum* (tomato)	*Ca*. Phytoplasma solani (bacterium)	Yes	*Candidatus* Phytoplasma solani	466	10625	111578
SRR7186379	*Glycine max* (soybean)	*Ca*. Phytoplasma sp. (bacterium)	Yes (different species)	*'Parthenium hysterophorus'* phyllody phytoplasma	502	4541	23497
SRR17253894	*Prunus pseudocerasus* (Chinese cherry)	Ca. Phytoplasma sp. (bacterium)	Yes (different species)	*Candidatus* Phytoplasma ziziphi	13	3154	76
SRR8003868	*Solanum sisymbriifolium* (sticky nightshade)	*Verticillium dahliae* (fungus)	Yes	*Verticillium dahliae*	19	3114	235401
				*Verticillium dahliae* VdLs.17	15357	7370	23971
				*Verticillium dahliae* JR2	446	4269	1159
SRR6760520	*Olea europaea* (olive)	*Verticillium dahliae* (fungus)	Yes	*Verticillium dahliae*	33	3819	1138562
				*Verticillium dahliae* VdLs.17	15413	8672	109293
				*Verticillium dahliae* JR2	489	17964	4196
SRR1525437	*Olea europaea* (olive)	*Verticillium dahliae* (fungus)	Yes	*Verticillium dahliae* VdLs.17	8	382	46
SRR6053344	*Gossypium hirsutum* (upland cotton)	*Verticillium dahliae* (fungus)	Yes	*Verticillium dahliae*	54	3908	282087
				*Verticillium dahliae* VdLs.17	15192	5044	26609
				*Verticillium dahliae* JR2	394	4307	1320
SRR7814393	*Malus domestica* (apple)	*Erwinia amylovora* (bacterium)	No	NA	NA	NA	NA
SRR13488408	*Capsicum annuum* (pepper)	*Xanthomonas campestris* pv. Vesicatoria (bacterium)	Yes (different species)	*Xanthomonas euvesicatoria*	2	2417	6
ERR2036424	*Triticum aestivum* (wheat)	*Xanthomonas translucens* (bacterium)	No	NA	NA	NA	NA
ILVO_Cpnnyn (SRR24305225)	*Chrysanthemum* × *morifolium* (florist’s daisy)	*Puccinia horiana* (fungus)	Yes	*Puccinia triticina*	105	1976	1010
				*Puccinia graminis* f. sp. tritici CRL 75–36-700–3	115	1362	265
				*Puccinia striiformis* f. sp. tritici	63	875	177
ILVO_Salix (SRR24305224)	*Salix alba* (white willow)	*Xylella fastidiosa* (bacterium)	No	NA	NA	NA	NA
ILVO_Daucu (SRR24305223)	*Daucus carota* (carrot)	*Ca*. Phytoplasma asteris (bacterium)	Yes (different species)	Aster yellows witches'-broom phytoplasma AYWB	26	2087	2694
AGS_feve (SRR24305222)	*Vicia faba* (broad bean)	*Ca*. Phytoplasma sp. Flavescence dorée (bacterium)	Yes (different species)	*Candidatus* Phytoplasma vitis	77	2739	1556
AGS_vigne (SRR24305220)	*Vitis vinifera* (grapevine)	downy mildew (fungus)	Yes (different species)	*Plasmopara viticola*	270	3687	40052
KIS_V3417 (SRR24305219)	*Daucus carota* (carrot)	*Alternaria* sp. (fungus)	Yes (different species)	*Alternaria solani*	3147	2442	38972
KIS_V3418 (SRR24305218)	*Solanum tuberosum* (potato)	*Alternaria* sp. (fungus)	Yes (different species)	*Alternaria solani*	298	911	4760
KIS_V3408 (SRR24305221)	*Solanum lycopersicum* (tomato)	*Phytophthora infestans* (oomycete)	Yes	*Phytophthora infestans*	25	2215	1265
				*Phytophthora infestans* T30-4	92	575	1062
KIS_V3408dup (SRR24305217)	*Solanum lycopersicum* (tomato)	*Phytophthora infestans* (oomycete)	Yes	*Phytophthora infestans*	103	2739	9963
				*Phytophthora infestans* T30-4	1920	2857	19252

**Fig. 3:**
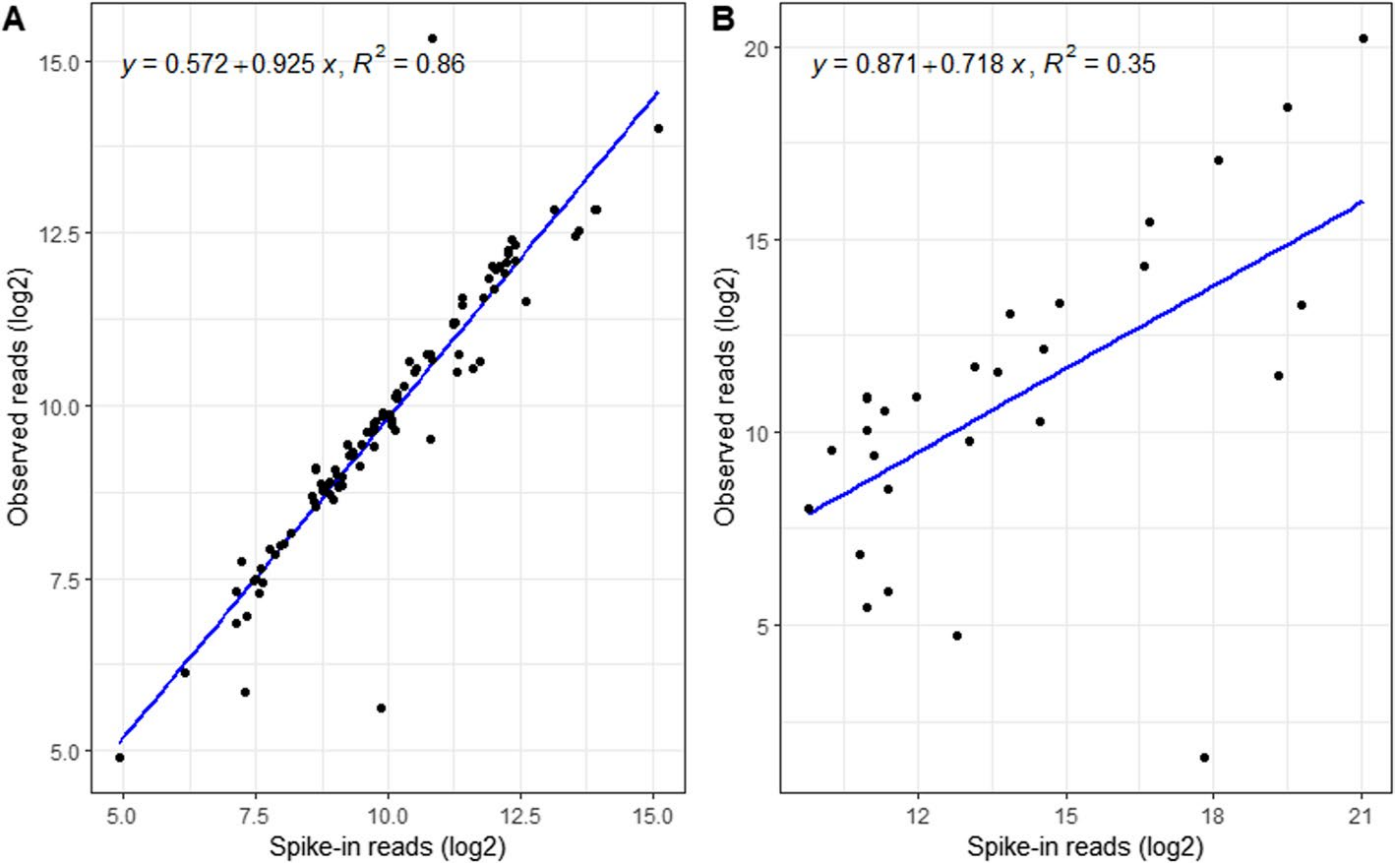
Comparison between spike-in and observed pathogen reads from simulated RNA-seq data. **A** 79 viruses/viroids from 12 crops. Observed reads were obtained by either mapping reads to a viral reference if viral contigs are annotated or are from Kraken2 classification. The high correlation between the spike-in and observed viral reads shows high detection ability of PhytoPipe. **B** 28 bacteria/fungi/oomycetes in 12 crops. Observed reads were obtained from Kraken2 classification

To further assess the ability of the pipeline to detect plant pathogens, twenty-four simulated RNA-seq datasets (12 with and 12 without pathogens) were generated using ART [74]. Twelve datasets comprising 10 or 20M host reads from 12 crop genomes (apple, cassava, citrus, grapevine, maize, peanut, potato, rice, rose, soybean, sweet potato, wheat) were generated using ART and the subsample function of seqtk (https://github.com/lh3/seqtk). Another twelve spike-in datasets were composed of a host, two to three fungi/bacteria/oomycetes, and six to eight viruses/viroids for each crop with different quantities. The virus/viroid reads in the spike-in samples ranged from 30 to 35,250 with a coverage from 2 to 300X, and fungi/bacteria/oomycetes reads ranged from 877 to 2,185,250 with a coverage from 1 to 10X (Additional file 1: Table S1). The spiked pathogens didn’t show up in the results of 11 host datasets as expected, except citrus endogenous pararetrovirus in the citrus host. In addition, four unexpected viruses were found in the other three host datasets: rice tungro bacilliform virus and citrus exocortis viroid in rice, caulimovirus sp. in sweetpotato, and begomovirus-associated DNA-III in cassava. These results show that the negative control sample has an important role in the analysis. PhytoPipe detected all 79 spiked viruses/viroids that were expected with the high level of correlation between the simulated and observed reads (the majority were mapped reads; classified reads were for viruses without contigs) (R^2^ equals to 0.86) (Fig. 3A). Viruses missed by the assembly-based method were detected by the Kraken2 classification method. In case of citrus endogenous pararetrovirus, the number of observed reads was double the number of spiked ones. All 13 bacteria/oomycetes pathogens and 13 out of 15 fungi were detected by PhytoPipe at the species level despite the low correlation (R^2^ equals to 0.35) between the simulated and observed classified reads (Fig. 3B). The spiked reads classified by Kraken2 varied from less than 1% to 95% of the original ones. Eight pathogens (six fungi and two bacteria) had less than 10% reads classified at the species level and one of the spiked fungi *Diplocarpon rosae* had just three reads classified to this species. These results showed that the coverage and the database are also the keys for RNA-seq to detect pathogens. Although viruses and viroids can be detected with less than 1000 reads due to their smaller genomes, fungi, bacteria, and oomycetes require high titer because of the large genome size. In summary, PhypoPipe can detect spiked microbes in the simulated data with a high level of accuracy. For the virus detection, TPR, FNR and FDR of the pipeline are 100%, 0%, and 2.4%, respectively. Two unexpected viruses, rice tungro bacilliform virus and citrus exocortis viroid were detected in both rice and spike-in rice datasets, are counted as false positives. For the detection of bacteria, fungi, and oomycetes, if only the same species are treated as true positives, TPR, FNR and FDR of the pipeline are 97%, 14%, and 40%, respectively. FDR is high because the simulated genome coverage (1 to 10X) is comparatively low and many reads are classified into the same family or genus, not into the same species.

## Discussion

To assess whether plant material is pathogen-free is critical for regulatory and biosecurity purposes. In contrast to the use of PCR and ELISA, which target a specific pathogen, RNA-seq can detect all potential pathogens in a plant sample. However, the results could be impacted by several variables, including the type of tissue used, the pathogen titer in the sample, the type of nucleic acid under analysis, the sequencing method employed, the type of analytical tools, and the reference databases used by each analytical tool. Virus detection pipelines can also vary greatly in their ability to detect known and novel viruses. For example, an assembly-based analysis could generate false-negative results when low titer viruses are present in the sample. This potentially high-risk scenario could be due in part to the low incidence of reads that does not allow the generation of sizeable contigs. Contrary to this, read classification-based methods can detect those viruses, but they could be below the threshold. On the other hand, to speed up the detection process, many virus detection pipelines only use viral databases. This could potentially result in some host sequences being annotated as part of a virus genome by blast if the threshold is not strict, or some host reads mapping to viruses for the reference-based mapping method if the tool parameters are less stringent. To address these limitations, we built PhytoPipe, which can integrate read classification, assembly-based annotation, and reference-based mapping methods for the detection of known plant pathogens as well as novel viruses. The possible known viruses and viroids are identified by the overlap of the results from read classification at the nucleotide level by Kraken2 and contig blastn against the viral nucleotide database. The selected candidates are further filtered by the viral reference genome coverage from the reference-based mapping and their consensus sequence annotations from blastn against NCBI nt database. Furthermore, PhytoPipe can identify possible new viruses by the overlap of the results between read classification at the protein level by Kaiju and contig Diamond blastx against the viral protein database. Moreover, PhytoPipe visualizes the sequence profile as Krona pie charts which the users can use to determine the presence of any pathogens (Fig. 2D).

When a method is used for the analysis of HTS data, a threshold is either set by the user or by the developer. There is a trade-off between the true positive rate and the false positive rate for different thresholds. The more stringent the threshold is, the higher could be the number of false negatives, and vice versa. For example, if > 100 reads is used as a read number threshold for the Kraken2 read classification in the sample SRX6762507 (Table 1), three apple viroids (hop stunt viroid, hop latent viroid, and apple hammerhead viroid) could result in false negatives. Furthermore if > 60% viral genome coverage, which is defined as the percentage of a viral genome/segment covered by reads, is used to filter viruses in the PhytoPipe report of SRX6762507, three false positive viruses (apricot latent virus, turnip vein-clearing virus, and ribgrass mosaic virus) can be classified as non-detectable. Therefore, the use of the most appropriate thresholds to reduce false positives and false negatives is critical for an accurate diagnostic. PhytoPipe combines three methods to minimize the detection of false outcomes. If a virus is found by both the classification and assembly-based annotation methods, and its genome coverage (from the read mapping method) is above 15%, PhytoPipe reports this virus as a positive. This viral genome coverage threshold could be low and cause more false positives. Moreover, PhytoPipe has a user-defined pathogen file, named monitorPathogen.txt, which can be modified based on the user's pathogens of interest, such as the nationwide pest priorities. In case these monitored pathogens are missed in the report to cause false negatives, they can be fished out from the Kraken2 result, even with a low read number support (e.g., < 100 read). However, these could have higher probabilities of being false positives. In this circumstance, the user’s knowledge about the pathogen is key to decide whether there is a need for validation or not. Since the pathogen titer is often variable and depends on various factors, such as biotic, abiotic, and methodology challenges to the sample, it is difficult to establish a general threshold that can be applied for all pathogens. The PhytoPipe user can then easily filter the output for all the potential organisms present in the sample using the report files. For example, when looking into the virome of a sample, mapped reads, viral genome coverage, and blast E-value in the report (report.txt) can be used for filtering. For bacteria and fungi, classified read number, contig number, and longest contig size in the summary files (sample.blastnt.summary.txt and sample.blastnr.summary.txt) can be used. Users can determine the present of a positive diagnostic based on their expertise and by the inclusion of negative controls and taxonomy information from PhytoPipe. For further regulatory action, a wet-lab validation should be required for pathogens identified by HTS to minimize the risk of reporting false positives. Moreover, the plant pathogens that are detected despite a low number of reads, plant pathologists may also need to use alternative methods such as PCR, for the confirmatory diagnostics.

A phytosanitary inspection of plant material not only discovers the presence of known pathogens in the sample but also ensures a thorough examination to conclude that the material is free of detectable pathogens. However, it is difficult to determine whether a plant material is clean without a negative control. An HTS report for a plant sample could have many organisms, such as a plant, insects, plant fungi or environmental fungi (e.g., from soil, water, or air), plant bacteria or environmental bacteria, fungal viruses (or mycoviruses), plant viruses, insect viruses, etc. The negative control datasets can efficiently help to filter out unrelated organisms. On the other hand, inaccurate read classifications (with a low read number) or inaccurate contig annotations (with a high blast E-value) are also in the report since similar sequences in the databases are used for classification or annotation, and NCBI nt and nr databases are not curated and frequently updated. With negative control datasets, reasonable thresholds can be set up and wrong annotations can be removed. On the contrary, the absence of negative control datasets could result in a higher number of false positives or even a wrong report for a sample. Therefore, the negative control datasets are needed for reducing errors in the analysis to generate a reliable result.

Ribosomal RNA removal is a key step during the library preparation process when using total RNA as the initial sample source for diagnostics. rRNA could take up to 30–50% of the reads in a sequenced library with inefficient rRNA removal whereas efficient removal of rRNA can lead to samples with less than 5% of the reads. The PhytoPipe ribosomal RNA removal step uses SILVA Eukaryote ribosomal RNA (18S and 28S) database to evaluate the library prep and remove possible host rRNAs. To evaluate whether this step impacts the microbe detection, two analyses of 12 bacteria RNA-seq data from the study by Haegeman et al. [15] were done using Kraken2. One analysis was for raw reads using rRNA database SILVA 138_1 SSU while the other one was for rRNA removed reads using NCBI nt database (PhytoPipe method). Our results showed that the rRNA removal step had no impact on bacterial pathogen detection (Additional file 2: Table S2). Surprisingly, more reads were classified using the PhytoPipe method as compared to the one without rRNA removal.

The available pipelines such as Kodoja [29], VirFind [23], VSD toolkit [24], VirusDetect [25], and Virtool [26] are limited to viral pathogen detection. To the best of our knowledge, PhytoPipe is the first pipeline that has the potential to detect possible microbial pathogens in a plant using RNA-seq data. Besides the wider scope of pathogen detection, PhytoPipe offers many unique features that other pipelines lack (Table 4). First, a *k*-mer-based read classification method in PhytoPipe can detect the viruses missed by assembly- and mapping-based methods. Second, PhytoPipe can remove and report the percentage of host ribosomal RNA reads in the sample that facilitates the quality assessment of the wet lab work. Third, PhytoPipe subtracts host reads using the *k*-mer method (based on the Kraken2 result) for all plants instead of host genome mapping for only plants having a complete host genome available. Fourth, PhytoPipe summarizes results from the comprehensive analysis as an HTML report (report.html) that helps users to determine the presence of possible pathogens in the sample. Lastly, the Snakemake workflow management system allows seamless integration and scaling of the pipelines to server, cluster, and cloud environments.

**Table 4:** Unique features of PhytoPipe in comparison with other pipelines

	PhytoPipe	Kodoja	Pathoscope	Virtool	VirFind	VSD toolkit	VirusDetect
Platform	Local Linux	Local Linux	Local Linux	Local Linux web GUI	Web	Web	Web/Local Linux
Workflow management	Snakemake	None	None	None	None	Yabi	None
Sequencing technique	RNA seq	RNA seq	RNA seq	RNA seq	RNA seq	small RNA seq	small RNA seq
Detection of known viruses	Yes	Yes	Yes	Yes	Yes	Yes	Yes
Novel virus discovery	Blastx + Kaiju	No	No	HMMER	Blastx	Blastx	Blastx
Detection of phytoplasma	Yes	No	Yes	No	No	No	No
Detection of bacteria	Yes	No	Yes	No	No	No	No
Detection of fungi	Yes	No	Yes	No	No	No	No
Read quality control	Yes	Yes	No	Yes	Yes	Yes	Yes
Host removal	*k*-mer based extraction	No	No	Host genome mapping	Host genome mapping	Host genome mapping	Host genome mapping
Ribosomal RNAs removal	Yes	No	No	No	No	No	No
Duplicates removal	Yes	No	No	No	No	No	No
Read classification	Yes	Yes	No	No	No	No	No
de novo assembly	Yes	No	No	Yes	Yes	Yes	Yes
Viral reference mapping	Yes	No	Yes	Yes	No	Yes	No
Sequence profile	Yes	No	No	No	No	No	No
Multiple samples	Yes	No	No	No	No	No	Yes

Despite our best effort to design a comprehensive phytopathogen detection method, PhytoPipe has a few limitations. First, the pipeline is mainly developed for the RNA sequencing data generated using the Illumina sequencing platform. However, it can be extended to other platforms after adjusting for a different platform-related set of tools. Second, the pipeline uses several different databases, which takes time, effort, and significant data storage space for initial construction and maintenance. Third, the pipeline can be used to identify the low titer viruses, which have a low read number supporting the output analysis. Such viruses could be false positives in the report, but this is a limitation found in all the pipelines investigated in this study. Fourth, the pipeline reports possible bacteria and fungi from classification and assembly-based annotation (see Table 1). The end-users of the results need to perform further validation of the results and use their knowledge of plant pathogens to make a regulatory decision. Lastly, only simulated datasets were used to evaluate PhytoPipe detection of all possible microbes in plant samples. This evaluation is still limited without large real datasets.

## Conclusions

PhytoPipe is a reliable and robust bioinformatic framework for detecting plant pathogens (bacteria (including phytoplasma), fungi, oomycetes, viroids, and viruses (including novel ones)) using RNA-seq data. Pathogens are identified with HTS read classification and assembly-based annotation methods and further validated with reference-based mapping for viruses. PhytoPipe combines different tools and databases to verify the findings from various angles. Although PhytoPipe is uniquely designed for plant pathogen discovery, it can also be used for the detection of other organisms. A summary HTML file includes metagenomic information from HTS read classification, contig blast annotation, and reference-based mapping for downstream analysis and visualization. An organized running folder keeps detailed information for the user to explore the run information and results. PhytoPipe is implemented using Snakemake, which can take advantage of multicore CPUs in a local, cluster, or cloud environment. The PhytoPipe docker image can be used on a Linux, Mac, or Windows system.

The source code for PhytoPipe is distributed under a BSD-3 license and is freely available at https://github.com/healthyPlant/PhytoPipe. Software documentation available at https://github.com/healthyPlant/PhytoPipe/wiki describes the pipeline's installation, usage, and testing using the published RNA-seq data from NCBI SRA.

### Supplementary Information

**Additional file 1. Table S1**: Overview of simulated RNA-seq data using plant genomes and spiked-in pathogens.

**Additional file 2. Table S2**: Comparison of bacterial pathogen detection performed by Kraken2 implemented in PhytoPipe using raw reads against SILVA rRNA database and rRNA removed reads against NCBI nt database.

## Data Availability

The PhytoPipe homepage and code can be found at https://github.com/healthyPlant/PhytoPipe. The documentation and test procedures are both available on GitHub at https://github.com/healthyPlant/PhytoPipe/wiki. PhytoPipe docker image is available on Docker hub at https://hub.docker.com/r/healthyplant/phytopipe. Availability and requirements—Project name: PhytoPipe. Project home page: https://github.com/healthyPlant/PhytoPipe. Operating system(s): Linux, Mac, Windows (Docker image only). Programming language: Python3, Bash and Snakemake. Other requirements: Python 3.6 or greater, Snakemake 5.13 or greater, and NCBI Blast v2.10 or greater. License: BSD-3. Any restrictions to use by non-academics: license needed.

## References

[1] MacDiarmid R, Rodoni B, Melcher U, Ochoa-Corona F, Roossinck M (2013). Biosecurity implications of new technology and discovery in plant virus research. PLoS Pathog.

[2] Martin RR, Constable F, Tzanetakis IE (2016). Quarantine regulations and the impact of modern detection methods. Annu Rev Phytopathol.

[3] Halewood M, Jamora N, Noriega IL, Anglin NL, Wenzl P, Payne T, Ndjiondjop M-N, Guarino L, Kumar PL, Yazbek M (2020). Germplasm acquisition and distribution by CGIAR genebanks. Plants.

[4] Stephen Smith TEN (2021). Mary Challender: Germplasm exchange is critical to conservation of biodiversity and global food security. Agron J.

[5] Martin RR, James D, Levesque CA (2000). Impacts of molecular diagnostic technologies on plant disease management. Annu Rev Phytopathol.

[6] Mumford R, Boonham N, Tomlinson J, Barker I (2006). Advances in molecular phytodiagnostics—new solutions for old problems. Eur J Plant Pathol.

[7] Costa LC, Hu XJ, Malapi-Wight M, O'Connell M, Hendrickson LM, Turner RS, McFarland C, Foster J, Hurtado-Gonzales OP (2022). Genomic characterization of silvergrass cryptic virus 1, a novel partitivirus infecting Miscanthus sinensis. Arch Virol.

[8] Gauthier MEA, Lelwala RV, Elliott CE, Windell C, Fiorito S, Dinsdale A, Whattam M, Pattemore J, Barrero RA (2022). Side-by-side comparison of post-entry quarantine and high throughput sequencing methods for virus and viroid diagnosis. Biology.

[9] Kumar LM, Foster JA, McFarland C, Malapi-Wight M (2018). First report of Barley virus G in switchgrass (*Panicum virgatum*). Plant Dis.

[10] Malapi-Wight M, Adhikari B, Zhou J, Hendrickson L, Maroon-Lango CJ, McFarland C, Foster JA, Hurtado-Gonzales OP (2021). HTS-based diagnostics of sugarcane viruses: seasonal variation and its implications for accurate detection. Viruses.

[11] Maree HJ, Fox A, Al Rwahnih M, Boonham N, Candresse T (2018). Application of HTS for routine plant virus diagnostics: state of the art and challenges. Front Plant Sci.

[12] Massart S, Candresse T, Gil J, Lacomme C, Predajna L, Ravnikar M, Reynard JS, Rumbou A, Saldarelli P, Skoric D (2017). A framework for the evaluation of biosecurity, commercial, regulatory, and scientific impacts of plant viruses and viroids identified by NGS technologies. Front Microbiol.

[13] Villamor DEV, Ho T, Al Rwahnih M, Martin RR, Tzanetakis IE (2019). High throughput sequencing for plant virus detection and discovery. Phytopathology.

[14] Espindola AS, Cardwell K, Martin FN, Hoyt PR, Marek SM, Schneider W, Garzon CD (2022). A step towards validation of high-throughput sequencing for the identification of plant pathogenic oomycetes. Phytopathology.

[15] Haegeman A, Foucart Y, De Jonghe K, Goedefroit T, Al Rwahnih M, Boonham N, Candresse T, Gaafar YZA, Hurtado-Gonzales OP, Kogej Zwitter Z (2023). Looking beyond virus detection in RNA sequencing data: lessons learned from a community-based effort to detect cellular plant pathogens and pests. Plants.

[16] Malapi-Wight M, Salgado-Salazar C, Demers JE, Clement DL, Rane KK, Crouch JA (2016). Sarcococca blight: use of whole-genome sequencing for fungal plant disease diagnosis. Plant Dis.

[17] Nizamani MM, Zhang Q, Muhae-Ud-Din G, Wang Y (2023). High-throughput sequencing in plant disease management: a comprehensive review of benefits, challenges, and future perspectives. Phytopathol Res.

[18] Massart S, Olmos A, Jijakli H, Candresse T (2014). Current impact and future directions of high throughput sequencing in plant virus diagnostics. Virus Res.

[19] FAO: Preparing to use high-throughput sequencing (HTS) technologies as a diagnostic tool for phytosanitary purposes. Commission on Phytosanitary Measures Recommendation; 2019. p. 8.

[20] EPPO. PM 7/151 (1) Considerations for the use of high throughput sequencing in plant health diagnostics. OEPP/EPPO Bull. 2022;52(3):619–42.

[21] Poretsky R, Rodriguez RL, Luo C, Tsementzi D, Konstantinidis KT (2014). Strengths and limitations of 16S rRNA gene amplicon sequencing in revealing temporal microbial community dynamics. PLoS ONE.

[22] Costa LC, Atha B, Hu X, Lamour K, Yang Y, O'Connell M, McFarland C, Foster JA, Hurtado-Gonzales OP (2022). High-throughput detection of a large set of viruses and viroids of pome and stone fruit trees by multiplex PCR-based amplicon sequencing. Front Plant Sci.

[23] Morgan XC, Huttenhower C (2012). Chapter 12: human microbiome analysis. PLoS Comput Biol.

[24] Adams IP, Fox A, Boonham N, Massart S, De Jonghe K (2018). The impact of high throughput sequencing on plant health diagnostics. Eur J Plant Pathol.

[25] Piombo E, Abdelfattah A, Droby S, Wisniewski M, Spadaro D, Schena L (2021). Metagenomics approaches for the detection and surveillance of emerging and recurrent plant pathogens. Microorganisms.

[26] Roossinck MJ (2012). Plant virus metagenomics: biodiversity and ecology. Annu Rev Genet.

[27] Kutnjak D, Tamisier L, Adams I, Boonham N, Candresse T, Chiumenti M, De Jonghe K, Kreuze JF, Lefebvre M, Silva G (2021). A primer on the analysis of high-throughput sequencing data for detection of plant viruses. Microorganisms.

[28] Massart S, Chiumenti M, Jonghe K, Glover R, Haegeman A, Koloniuk I, Kominek P, Kreuze J, Kutnjak D, Lotos L (2019). Virus detection by high-throughput sequencing of small RNAs: large-scale performance testing of sequence analysis strategies. Phytopathology.

[29] Wu QF, Luo YJ, Lu R, Lau N, Lai EC, Li WX, Ding SW (2010). Virus discovery by deep sequencing and assembly of virus-derived small silencing RNAs. Proc Natl Acad Sci USA.

[30] Kim NY, Lee HJ, Kim HS, Lee SH, Moon JS, Jeong RD (2021). Identification of plant viruses infecting pear using RNA sequencing. Plant Pathol J.

[31] Kimbrel JA, Di YM, Cumbie JS, Chang JH (2011). RNA-Seq for plant pathogenic bacteria. Genes.

[32] Xu GR, Strong MJ, Lacey MR, Baribault C, Flemington EK, Taylor CM (2014). RNA CoMPASS: a dual approach for pathogen and host transcriptome analysis of RNA-Seq datasets. PLoS ONE.

[33] Lebas B, Adams I, Al Rwahnih M, Baeyen S, Bilodeau Guillaume J, Blouin AG, Boonham N, Candresse T, Chandelier A, De Jonghe K (2022). Facilitating the adoption of high-throughput sequencing technologies as a plant pest diagnostic test in laboratories: a step-by-step description. EPPO Bull.

[34] Hodge BA, Paul PA, Stewart LR (2020). Occurrence and high-throughput sequencing of viruses in Ohio wheat. Plant Dis.

[35] Al Rwahnih M, Daubert S, Golino D, Islas C, Rowhani A (2015). Comparison of next-generation sequencing versus biological indexing for the optimal detection of viral pathogens in grapevine. Phytopathology.

[36] Bester R, Cook G, Breytenbach JHJ, Steyn C, De Bruyn R, Maree HJ (2021). Towards the validation of high-throughput sequencing (HTS) for routine plant virus diagnostics: measurement of variation linked to HTS detection of citrus viruses and viroids. Virol J.

[37] Rott M, Xiang Y, Boyes I, Belton M, Saeed H, Kesanakurti P, Hayes S, Lawrence T, Birch C, Bhagwat B (2017). Application of next generation sequencing for diagnostic testing of tree fruit viruses and viroids. Plant Dis.

[38] Karavina C, Ibaba JD, Gubba A (2020). High-throughput sequencing of virus-infected *Cucurbita pepo* samples revealed the presence of Zucchini shoestring virus in Zimbabwe. BMC Res Notes.

[39] Bharti R, Grimm DG (2021). Current challenges and best-practice protocols for microbiome analysis. Brief Bioinform.

[40] Sczyrba A, Hofmann P, Belmann P, Koslicki D, Janssen S, Droge J, Gregor I, Majda S, Fiedler J, Dahms E (2017). Critical assessment of metagenome interpretation—a benchmark of metagenomics software. Nat Methods.

[41] Hong CJ, Manimaran S, Shen Y, Perez-Rogers JF, Byrd AL, Castro-Nallar E, Crandall KA, Johnson WE (2014). PathoScope 2.0: a complete computational framework for strain identification in environmental or clinical sequencing samples. Microbiome.

[42] Norouzi-Beirami MH, Marashi SA, Banaei-Moghddam AM, Kavousi K (2021). CAMAMED: a pipeline for composition-aware mapping-based analysis of metagenomic data. NAR Genomics Bioinform.

[43] Ho T, Tzanetakis IE (2014). VirFind: an online bioinformatics tool for plant virus detection and discovery. Phytopathology.

[44] Barrero RA, Napier KR, Cunnington J, Liefting L, Keenan S, Frampton RA, Szabo T, Bulman S, Hunter A, Ward L (2017). An internet-based bioinformatics toolkit for plant biosecurity diagnosis and surveillance of viruses and viroids. BMC Bioinform.

[45] Zheng Y, Gao S, Padmanabhan C, Li RG, Galvez M, Gutierrez D, Fuentes S, Lin KS, Kreuze J, Fei ZJ (2017). VirusDetect: an automated pipeline for efficient virus discovery using deep sequencing of small RNAs. Virology.

[46] Menzel P, Ng KL, Krogh A (2016). Fast and sensitive taxonomic classification for metagenomics with Kaiju. Nat Commun.

[47] Wood DE, Lu J, Langmead B (2019). Improved metagenomic analysis with Kraken 2. Genome Biol.

[48] Baizan-Edge A, Cock P, MacFarlane S, McGavin W, Torrance L, Jones S (2019). Kodoja: a workflow for virus detection in plants using k-mer analysis of RNA-sequencing data. J Gen Virol.

[49] Koster J, Rahmann S (2012). Snakemake—a scalable bioinformatics workflow engine. Bioinformatics.

[50] Merkel D (2014). Docker: lightweight linux containers for consistent development and deployment. Linux J.

[51] Yilmaz P, Parfrey LW, Yarza P, Gerken J, Pruesse E, Quast C, Schweer T, Peplies J, Ludwig W, Glockner FO (2014). The SILVA and "All-species Living Tree Project (LTP)" taxonomic frameworks. Nucleic Acids Res.

[52] Kopylova E, Noe L, Touzet H (2012). SortMeRNA: fast and accurate filtering of ribosomal RNAs in metatranscriptomic data. Bioinformatics.

[53] Bolger AM, Lohse M, Usadel B (2014). Trimmomatic: a flexible trimmer for Illumina sequence data. Bioinformatics.

[54] BBTools suite. https://sourceforge.net/projects/bbmap/.

[55] FastQC: FastQC: a quality control tool for high throughput sequence data; 2015. http://www.Rbioinformaticsbabrahamacuk/projects/fastqc/.

[56] Ewels P, Magnusson M, Lundin S, Kaller M (2016). MultiQC: summarize analysis results for multiple tools and samples in a single report. Bioinformatics.

[57] Garcia BJ, Simha R, Garvin M, Furches A, Jones P, Gazolla JGFM, Hyatt PD, Schadt CW, Pelletier D, Jacobson D (2021). A k-mer based approach for classifying viruses without taxonomy identifies viral associations in human autism and plant microbiomes. Comput Struct Biotec.

[58] Ren J, Ahlgren NA, Lu YY, Fuhrman JA, Sun FZ (2017). VirFinder: a novel k-mer based tool for identifying viral sequences from assembled metagenomic data. Microbiome.

[59] Ondov BD, Bergman NH, Phillippy AM (2011). Interactive metagenomic visualization in a Web browser. BMC Bioinform.

[60] KrakenTools: Kraken Tools; 2021. https://githubcom/jenniferlu717/KrakenTools.

[61] Bankevich A, Nurk S, Antipov D, Gurevich AA, Dvorkin M, Kulikov AS, Lesin VM, Nikolenko SI, Pham S, Prjibelski AD (2012). SPAdes: a new genome assembly algorithm and its applications to single-cell sequencing. J Comput Biol.

[62] Grabherr MG, Haas BJ, Yassour M, Levin JZ, Thompson DA, Amit I, Adiconis X, Fan L, Raychowdhury R, Zeng QD (2011). Full-length transcriptome assembly from RNA-Seq data without a reference genome. Nat Biotechnol.

[63] Gurevich A, Saveliev V, Vyahhi N, Tesler G (2013). QUAST: quality assessment tool for genome assemblies. Bioinformatics.

[64] Camacho C, Coulouris G, Avagyan V, Ma N, Papadopoulos J, Bealer K, Madden TL (2009). BLAST plus: architecture and applications. BMC Bioinform.

[65] Buchfink B, Reuter K, Drost HG (2021). Sensitive protein alignments at tree-of-life scale using DIAMOND. Nat Methods.

[66] Bigot T, Temmam S, Perot P, Eliot M (2020). RVDB-prot, a reference viral protein database and its HMM profiles. F1000Research.

[67] Lefkowitz EJ, Dempsey DM, Hendrickson RC, Orton RJ, Siddell SG, Smith DB (2018). Virus taxonomy: the database of the International Committee on Taxonomy of Viruses (ICTV). Nucleic Acids Res.

[68] Li H. Aligning sequence reads, clone sequences and assebly contigs using BWA-MEM. arXiv 2013, 1303(3997v2).

[69] Li H, Handsaker B, Wysoker A, Fennell T, Ruan J, Homer N, Marth G, Abecasis G, Durbin R, Proc GPD (2009). The sequence Alignment/Map format and SAMtools. Bioinformatics.

[70] Hunter JD (2007). Matplotlib: a 2D graphics environment. Comput Sci Eng.

[71] Danecek P, Bonfield JK, Liddle J, Marshall J, Ohan V, Pollard MO, Whitwham A, Keane T, McCarthy SA, Davies RM (2021). Twelve years of SAMtools and BCFtools. Gigascience.

[72] Wright AA, Cross AR, Harper SJ (2020). A bushel of viruses: identification of seventeen novel putative viruses by RNA-seq in six apple trees. PLoS ONE.

[73] Tamisier LH, Annelies A, Foucart Y, Fouillien N, Al Rwahnih M, Buzkan N, Candresse T, Chiumenti M, De Jonghe K, Lefebvre M, Margaria P, Reynard JS, Stevens K, Kutnjak D, Massart S (2021). Semi-artificial datasets as a resource for validation of bioinformatics pipelines for plant virus detection. Peer Community J.

[74] Huang W, Li L, Myers JR, Marth GT (2012). ART: a next-generation sequencing read simulator. Bioinformatics.

